# RBM10: Harmful or helpful‐many factors to consider

**DOI:** 10.1002/jcb.26644

**Published:** 2018-01-19

**Authors:** Julie J. Loiselle, Leslie C. Sutherland

**Affiliations:** ^1^ Health Sciences North Research Institute (HSNRI) Sudbury Ontario Canada

**Keywords:** RBM5, RBM10, regulation, RNA binding proteins, SMN, TARP

## Abstract

*RBM10* is an RNA binding motif (RBM) protein expressed in most, if not all, human and animal cells. Interest in RBM10 is rapidly increasing and its clinical importance is highlighted by its identification as the causative agent of TARP syndrome, a developmental condition that significantly impacts affected children. RBM10's cellular functions are beginning to be explored, with initial studies demonstrating a tumor suppressor role. Very recently, however, contradictory results have emerged, suggesting a tumor promoter role for RBM10. In this review, we describe the current state of knowledge on RBM10, and address this dichotomy in RBM10 function. Furthermore, we discuss what may be regulating RBM10 function, particularly the importance of *RBM10* alternative splicing, and the relationship between RBM10 and its paralogue, RBM5. As RBM10‐related work is gaining momentum, it is critical that the various aspects of RBM10 molecular biology revealed by recent studies be considered moving forward. It is only if these recent advances in RBM10 structure and function are considered that a clearer insight into RBM10 function, and the disease states with which RBM10 mutation is associated, will be gained.

## 1 INTRODUCTION

RNA binding proteins (RBPs) are a large and broad class of proteins that regulate all aspects of RNA metabolism.^1^ RBPs are, therefore, involved in regulating the nature, quantity and functionality of gene expression products. The interest in one particular RBP, RNA binding motif protein 10 (RBM10), has increased substantially in the last few years, with a greater than 50% increase in RBM10‐related PubMed‐indexed publications (ie, 34 manuscripts) since 2013.

*RBM10* maps to the X‐chromosome at position p11.23.^2^, ^3^ It was first cloned from human bone marrow in 1995, within a collection of unidentified cDNAs.^4^ In 1996, RBM10 (as S1‐1) cDNA was cloned from rat liver in a targeted attempt to characterize a distinct subset of nuclear hnRNA‐associated proteins.^5^ The full‐length *RBM10* transcript is approximately 3.5 kb long, divided into 24 exons, and translated into a protein of 930 amino acids.^6^ *RBM10* is expressed in most, if not all, cells types (Gene Cards data), though one *RBM10* allele is silenced in somatic female cells by X chromosome inactivation.^2^, ^3^ A requirement for *RBM10* expression during development is evidenced by studies showing that loss‐of‐function mutations are the cause of TARP syndrome, an abnormal developmental syndrome usually resulting in the affected child's death before or soon after birth.^6^, ^7^, ^8^ *RBM10* mutations are also observed in a number of cancer types.^9^, ^10^, ^11^, ^12^, ^13^, ^14^, ^15^ The association of *RBM10* mutation with disease states is not surprising as altered RBP expression and/or function is associated with a wide spectrum of diseases, most being of neurological, muscular, sensory or neoplastic origin.^16^

In line with its mutational status in certain cancers, are studies that have demonstrated tumor suppressor‐associated roles for RBM10. For instance, many initial functional studies associated *RBM10* expression with increased apoptosis,^17^ decreased cell proliferation,^18^ decreased colony formation,^19^ and decreased xenograft tumor growth.^20^ Counterintuitively, however, very recent studies have suggested a tumor promoter role for RBM10, and associated *RBM10* expression with additional processes and mechanisms of action.^21^, ^22^ This functional dichotomy highlights the importance of not only gaining a deeper understanding of the downstream consequences of RBM10 expression, but identifying the mechanisms responsible for regulating RBM10 itself. Aspects of RBM10 regulation that have been identified, but that require further investigation include: (1) the alternative splicing of *RBM10*; (2) autoregulation of *RBM10* at multiple levels; and (3) regulation of *RBM10* by another, very homologous, RBP, RBM5.

In this review, we first describe recent advances regarding RBM10's structure and mechanism of action. We then address the dichotomy in RBM10 function, and discuss the factors by which it may be regulated. As publication of RBM10 studies is rapidly accelerating, and outcomes relating to RBM10 research can have clinical relevance, this review serves to highlight important aspects of previous RBM10‐related studies that should be taken into account in future experimental endeavors.

### 1.1 RBM10 primary structure

**Figure 1:**
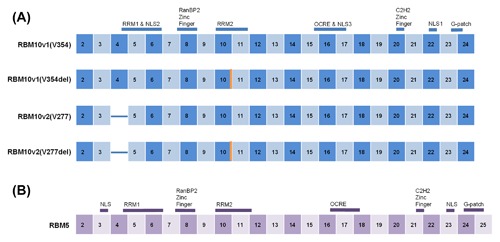
Selected RBM10 and RBM5 consensus functional motifs. Translated RBM10 (A) and RBM5 (B) exons are represented by boxes. Numbers indicated within a box designate the exon it represents within the corresponding transcript. Box size does not represent exon length. Location of RBM10 and RBM5 consensus functional motifs are indicated by the thick line above the corresponding box(es), and the type of motif is indicated. NLS refers to nuclear localization signal. Principal RBM10 alternative splice variants are also included (A), with the orange line representing the absence of a GTG RNA triplet at that location (end of exon 10)

Primary structure is used to identify consensus functional motifs, and thus predict the functional characteristics of a translated protein. Coded within RBM10 are a number of different consensus functional motifs. These include; two RNA Recognition Motif (RRM) domains, an OCRE domain, a G‐patch domain, a C2H2‐type zing finger domain, a RanBP2‐type zinc finger domain, and three nuclear localization signals (NLSs) (NLS2 and NLS3 are within the RRM1 and OCRE regions, respectively) (Figure 1A).^5^, ^23^, ^24^ The functionality associated with many of these consensus motifs within RBM10 is beginning to be examined. For example, the three‐dimensional structure of the RBM10 OCRE‐containing globular domain can influence the interaction between RBM10 and other molecules, particularly spliceosomal complex proteins.^25^ Furthermore, RBM10 NLSs can work cooperatively to influence nuclear localization of RBM10.^24^, ^26^ In terms of RNA binding, the RBM10 RanBP2‐type zinc finger domain, and both RRM domains can bind RNA; however, while the RBM10 RanBP2‐type zinc finger and RRM1 domains can act together to recognize and bind specific RNA consensus sequences, the RBM10 RRM2 domain is structurally independent from both the RanBP2‐type zinc finger and RRM1 domains, and can bind independently.^27^, ^28^ Taken together, determining how each RBM10 consensus functional motif contributes to the functionality of RBM10 will enable stronger predictions regarding the impact of *RBM10* mutations on RBM10 function.

### 1.2 RBM10 is more than a regulator of alternative splicing

As predicted by the consensus functional motifs in its primary sequence, RBM10 is a regulator of alternative splicing. This was first demonstrated in 2013/2014,^19^, ^29^, ^30^, ^31^ and has since been confirmed by a number of groups.^18^, ^20^, ^22^, ^32^ For instance, RBM10 is involved in the alternative splicing of pre‐mRNA from *NUMB*,^20^ *FAS*,^31^ *Dlg4*,^30^ and *SMN2*.^32^ RBM10 has even been shown to promote alternative splicing‐coupled nonsense‐mediated mRNA decay (AS‐NMD) of its own pre‐mRNA and that of *RBM5*.^33^ The interaction of RBM10 with components of spliceosomal complexes also supports a role for RBM10 as a regulator of alternative splicing.^34^, ^35^, ^36^

Multiple aspects of RNA metabolism can be influenced by one particular RBP, and it is becoming clear that this is the case for RBM10. For instance, although most early studies focused on the role of RBM10 as a regulator of alternative splicing, some research groups identified additional RNA regulatory roles. The first demonstration of a distinctly non‐splicing‐related role for RBM10 was a report that the rat equivalent of RBM10, S1‐1, bound the 3′UTR of the angiotensin receptor type 1 (*AT‐1*) transcript and increased transcript stability: this stabilization ultimately led to downregulation of *AT‐1* transcription.^37^ Since the rat and human RBM10 amino acid sequences share 97% homology, similar functionality is predicted for human RBM10.^23^ In support of this prediction, our group recently identified a number of RBM10 RNA targets that are involved in numerous aspects of the control of gene expression.^21^ RBM10 is therefore involved in at least three different mechanisms (pre‐mRNA splicing, mRNA stabilization, and mRNA transcription) related to RNA expression.

In addition, RBM10 is involved in a number of other cellular processes that are not at all, or not directly, related to RNA metabolism. These other cellular processes were identified through various protein interaction studies. For instance, RBM10 interacts with the FilGAP protein to control FilGAP localization and function^38^: RBM10 is thus involved in regulating cell structure and spreading. RBM10 also interacts with the 2A‐DUB deubiquitinase protein complex, which participates in histone modifications and thus regulates gene transcription^39^: RBM10 may, therefore, be involved in the epigenetic regulation of gene transcription through changes in the posttranslational modification of histones. Taken together, these studies show that RBM10 is involved in a number of different mechanisms that influence more than RNA metabolism. Future studies involving RBM10 should, therefore, recognize the limitations of focusing too narrowly when examining RBM10 mechanism and function.

To note, a number of studies have attempted to identify consensus RBM10 binding sequences within RBM10 target RNAs, an outcome that would enable prediction of additional RBM10 RNA targets and the potential effects of RBM10 binding. CLIP‐Seq, PAR‐CLIP, and iCLIP were used in these target binding sequence studies,^19^, ^22^, ^29^, ^40^ and a summary of the RBM10 consensus binding sequences determined by these studies is presented in Table 1. To note, the degree of similarity between the RBM10 consensus binding sequences in the RBM10 target RNAs identified to date is minimal, a finding that may be due to differences in the target identification technique, cell type and/or specific protein isoform used in each study. RNA target identification by RBM10 may thus be dependent on a number of factors, which remain to be elucidated.

**Table 1:** Summary of consensus RNA binding sequences for RBM10

Experimental technique	Consensus binding sequence(s)	Reference
RNA homopolymer beads	poly(U) & poly(G) > poly(C) > poly(A)	5
CLIP‐Seq	CUCUGAACUC CGAUCCCU	19
PAR‐CLIP and Discover computational tool^a^	Exonic sequence: GAAGA UGGA UCUUCA Intronic sequence: UUUCU CACCGUGG	29, 40
PAR‐CLIP and HOMER software^a^	UGUGGACA	28, 29
iCLIP	TCCAA CCAAA CCCCA	22
Fluorescence anisotropy titration and chemical shift pertubations (only with RanBP2 zinc finger domain of RBM10)	AGGUAA	27
RNAcompete (only with RanBP2 zinc finger and RRM1 domain of RBM10)	UGUGGA	28
Scaffold independent analysis (only with RRM2 domain of RBM10)	CCNC	28

Underlined sequence indicates core motif.

^a^ PAR‐CLIP study by Wang et al. did not publish any consensus binding sequences. Studies by Maaskola & Rajewsky and Collins et al. applied different computation tools to the PAR‐CLIP data from Wang et al. to output potential RBM10 consensus binding sequences.

## 2 THE CONTRASTING EFFECTS OF RBM10 EXPRESSION

The majority of studies relating to the downstream effects associated with changes in *RBM10* expression have centered on roles in the promotion of cell cycle arrest and apoptosis, largely due to *RBM10*'s homology to *RBM5*, an established apoptosis modulator.^23^ The first functional study correlated *RBM10* expression with decreased cell proliferation and increased apoptosis in hypertrophic primary chondrocytes.^41^ In 2012, our group confirmed that RBM10 promoted apoptosis in two human cancer cell lines, and identified a positive correlation between *RBM10* expression and TNFα transcription.^42^ Since then, the roles of RBM10 as a promoter of apoptosis^17^ and an inhibitor of proliferation^18^, ^19^, ^20^ have been confirmed in a number of studies. In addition, correlational studies from human tissues support an apoptosis‐promoting role for RBM10, as *RBM10* mRNA expression in breast cancer samples correlated with increased mRNA expression of BAX, a proapoptotic protein, and TP53, a tumor suppressor protein with transcriptional activity.^43^ Unexpectedly, however, the latter study also correlated *RBM10* expression with increased mRNA expression of *VEGF*, a potent promoter of angiogenesis.^43^

Our very recent work took a broader look at RBM10 function by using next‐generation sequencing to identify genes differentially expressed following modulation of *RBM10* expression levels.^21^ Unexpectedly, but using an *RBM5*‐null small cell lung cancer cell line, the results suggested that RBM10 promotes many transformation‐ and hypoxia‐associated processes and events. Specifically, knockdown of *RBM10* expression induced changes in gene expression predicted to reduce glycolysis, epithelial to mesenchymal transition (EMT) and angiogenesis − potentially by direct regulation of cell metabolism, specifically oxidative phosphorylation. Many recent studies support those findings: (1) the association of RBM10 with FilGAP,^38^ a regulator of cell spreading, supports a role for RBM10 in the regulation of EMT; (2) the phosphorylation of RBM10 by c‐SRC, and consequent involvement in the PDGF signaling pathway,^44^ supports a role for RBM10 in the promotion of angiogenesis; (3) the positive correlation between the mRNA expression of *RBM10* and *VEGF*,^43^ an important angiogenesis promoter, also supports a role for *RBM10* in the regulation of angiogenesis; and (4) the association of RBM10 with neurodegenerative disorders,^19^, ^21^ many of which involve impairment of oxidative phosphorylation, supports a role for RBM10 in the regulation of this aspect of cellular metabolism.

In fact, a pro‐transformatory role for RBM10 was previously demonstrated in embryonic stem cells and mouse mandibular cells, where knockdown of RBM10 was associated with decreased cell growth,^22^ suggesting that expression of RBM10 is associated with increases in cell growth. Furthermore, *RBM10* knockdown in neuronal cells increased caspase activation induced by staurosporine,^45^ suggesting that expression of RBM10 impedes apoptosis. This functional RBM10 dichotomy was recognized by Rodor et al^21^ who noted RBM10 may have cell type and/or species specific roles, but was directly tackled recently by our group: we proposed a working model that describes how the regulation of RBM10 expression determines function, whether tumor suppressive or tumor promoting.^21^ Notably, this working model contains many hypotheses that remain to be tested. Understanding the mechanisms that regulate *RBM10* expression and function would help to predict the impact of *RBM10* mutation on cellular processes and thus potential patient outcomes in a disease such as cancer.

## 3 REGULATION OF RBM10

**Figure 2:**
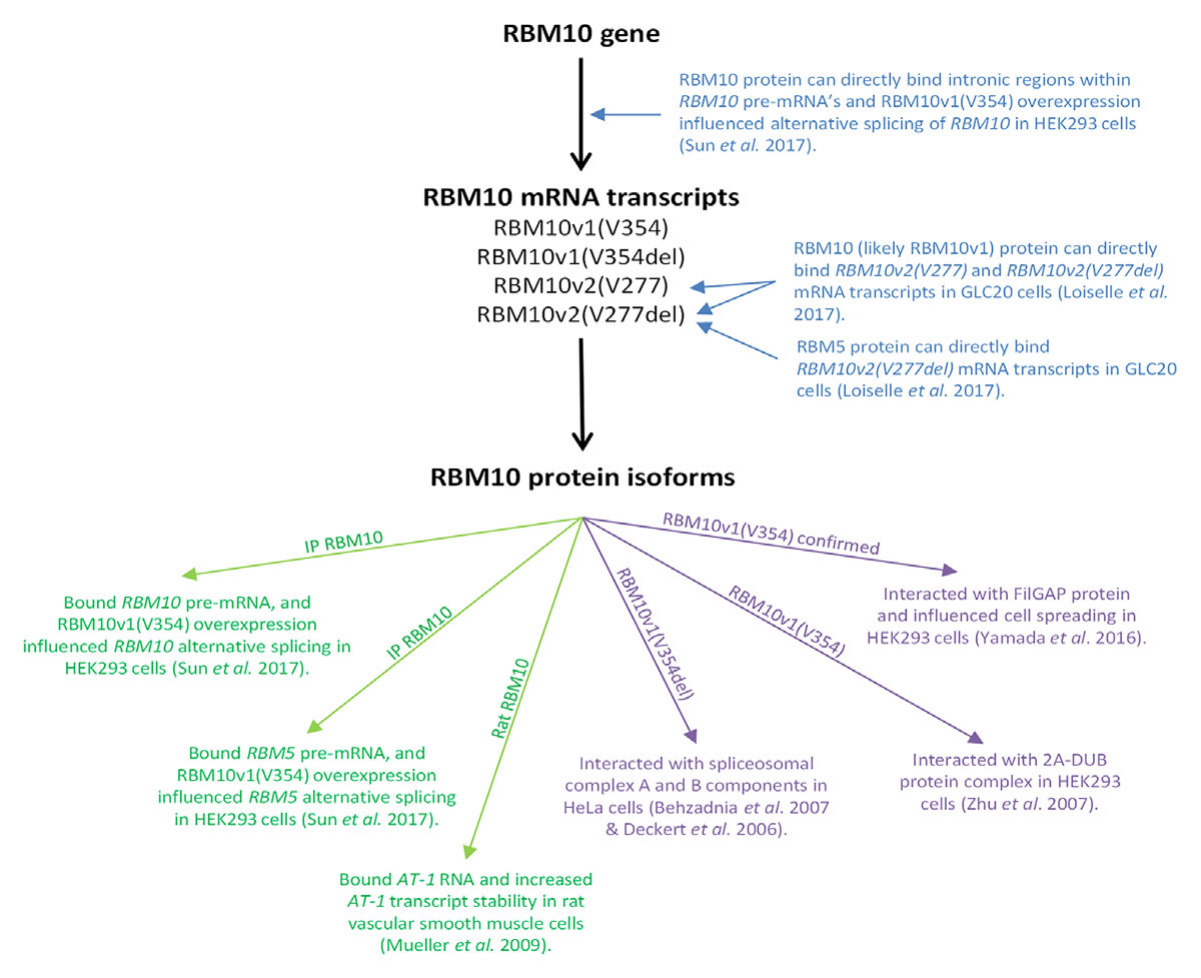
RBM10 interacting factors. Factors demonstrated to interact with RBM10 RNA (blue) and RBM10 protein (RNA factors in green, and protein factors in purple) are indicated. Established functional consequences of the interactions are indicated (if known). Text on arrows indicates which RBM10 isoform was involved in the study (as indicated by the referenced manuscript's published NCBI accession number or GenBank ID, when available). IP RBM10 indicates that RBM10‐interacting factors were identified by immunoprecipitation of RBM10 with an antibody with an immunogen sequence more homologous to RBM10v1 than RBM10v2 (the last 14 amino‐acids of its 81 amino acid immunogen sequence were not specific for RBM10v2) (Sigma HPA034972)

RBM10 mutation has particular relevance to TARP syndrome,^6^, ^8^ and may have relevance to spinal muscular atrophy (SMA).^32^ Unfortunately, actual changes in RBM10 expression have not been rigorously examined in either of these diseases. The fact that no significant changes in RBM10 expression have been identified in screens of other disease states likely accounts for the fact that the regulation of *RBM10* expression and function has, to date, been studied very little. What is known is that posttranslationally, RBM10 can be phosphorylated by c‐Src,^44^ an event that may influence RBM10 localization and function; upregulation of a Src family tyrosine kinase induced translocation of RBM10 from the nucleus to the cytoplasm, which was required for RBM10's interaction with FilGAP and consequent regulation of cell spreading.^38^ In addition, several recent studies have identified co‐ and post‐transcriptional RBM10‐regulatory mechanisms that may have a significant influence on RBM10 function: (1) alternative splicing of *RBM10*; (2) autoregulation of *RBM10* expression; and (3) regulation of RBM10 by the homologous RBP, RBM5. These specific regulatory mechanisms, as well as their potential functional consequences, are summarized in Figure 2, and described below in order to highlight how their consideration could impact the results of future RBM10‐related studies.

### 3.1 Alternative splicing of RBM10

Like 95% of multi‐exon containing transcripts,^46^ *RBM10* can be alternatively spliced. The main *RBM10* alternative splice variant, *RBM10v2*, is produced by alternatively splicing the fourth exon from *RBM10*, resulting in a protein of 853 amino acids.^6^, ^23^ As depicted in Figure 1A, the alternative splicing of exon four of *RBM10* alters the sequence of RBM10 RRM1, and thus, likely, it's RNA binding characteristics.

Recently, a comprehensive analysis of the alternative splicing of *RBM10* showed that both *RBM10v1* and *RBM10v2* have an additional splice variant that differs by only one RNA triplet, GTG (Figure 1B).^47^ This triplet is located at the very end of *RBM10v1* exon ten and encodes a valine residue. *RBM10* thus has four main alternative splice variants: (1) *RBM10v1(V354)*, the longest RBM10 transcript; (2) *RBM10v1(V354del)*, which lacks the GTG RNA triplet at the end of exon ten; (3) *RBM10v2(V277)*, which lacks the exon 4 of *RBM10v1*; and (4) *RBM10v2(V277del)*, which lacks the exon 4 of *RBM10v1*, and the GTG RNA triplet at the end of *RBM10v1* exon ten. Importantly, the valine residue coded by the alternatively spliced GTG triplet is located in RBM10's second RRM domain, at the beginning of its second α‐helix. Two studies have predicted the impact of the presence or absence of this valine residue on RBM10 structure and function, but with contrasting results. Firstly, Tessier et al,^47^ using the NMR structure of the RBM10 RRM2, predicted that the presence of valine inhibits alpha‐helix formation in RBM10 RRM2 and correlated this change in +/− GTG *RBM10* alternative splicing with alterations in RBM10 function. Secondly, Hernandez et al,^20^ using the solution structure of the RBM5 RRM2, predicted that the alternative valine residue within RBM10 was just outside of the RBM10 RRM2 alpha‐helix, on the opposite side of the RNA binding surface, and consequently that the presence or absence of valine would have little impact on RBM10 function. Interestingly, an earlier manuscript by Bechara et al^19^ showed that mutation of this alternatively spliced valine residue to glutamic acid did alter RBM10 function, supporting the notion that the alternative splicing of this codon could have functional implications for RBM10. In addition, our group recently showed that RBM5 can distinguish between these +/− GTG *RBM10* splice variants; RBM5 specifically bound only *RBM10v2(V277del)*.^21^ Taken together, these results suggest that the alternative splicing of *RBM10* results in changes in protein tertiary structure that either directly (eg, +/− valine in the RRM domain) or indirectly (eg, RBM10v1 versus RBM10v2) influence function.

Future studies should, therefore, consider the *RBM10* splice variant or isoform expression profile in the cell types used, prior to undertaking functional assays related to RBM10. Furthermore, it is essential to specify which particular variants or isoforms are being overexpressed or knocked down in, and which are able to be detected by, each experimental assay. This aspect of variant identification is almost always overlooked in RBM10‐related studies. Future studies that consider the expression and function of each RBM10 isoform would also be more accurate and informative. This is highlighted in Figure 2, which demonstrates that very little is known regarding RBM10‐splice variant interacting factors. In light of the fact that different isoforms may have opposing functions,^21^, ^47^ the ratio of the various RBM10 isoforms in a patient could be of predictive significance for disease incidence and/or progression.

### 3.2 Autoregulation of RBM10

It was very recently demonstrated that RBM10 is capable of binding its own pre‐mRNA thereby affecting alternative splicing and ultimately promoting its own nonsense‐mediated decay.^33^ Specifically, in HEK293 cells, RBM10 protein bound *RBM10* pre‐mRNA within the 5′‐splice sites of introns 6 and 12, resulting in increased levels of the *RBM10* exon 6 or exon 12 skipped variant. These exon 6 or exon 12 lacking variants are targets for NMD, and the authors demonstrated that overexpression of RBM10 ultimately negatively influenced both *RBM10* mRNA and protein levels. Thus, the potentially important functional consequences of *RBM10* alternative splicing, described above, can involve a level of co‐transcriptional self‐regulation. Of note, although not addressed by the authors, based on the NCBI Reference Sequence number provided, the *RBM10* variant overexpressed in this study was *RBM10v1(V354)*.

Interestingly, another very recently published study also showed that RBM10 protein is capable of binding its own mRNA, and possibly regulating its own translation. Using an RNA immunoprecipitation and next‐generation sequencing technique, RBM10 protein was shown to specifically bind the RNA of both *RBM10v2* variants: *RBM10v2*(*V277*) and *RBM10v2*(*V277del*).^21^ As the GLC20 cells used in this study almost exclusively expressed RBM10v1 at the protein level, it was most likely RBM10v1 that was interacting with the *RBM10v2* RNA. Strikingly, although RBM10v1 protein levels were substantially higher than RBM10v2 protein levels, mRNA expression levels of *RBM10v1* and *RBM10v2* were very similar, suggesting that the interaction of RBM10v1 protein with *RBM10v2* RNA is integral to an RBM10 translational self‐regulation mechanism, at least in GLC20 cells.^21^ Of note, in the study by Sun et al^33^ described above, RBM10v1 overexpression was followed by decreased RBM10v2 expression at both the mRNA and protein levels, suggesting that multiple levels of RBM10 self‐regulation exist in HEK293 cells. Taken together, these findings suggest that RBM10v1 is capable of regulating *RBM10v2* expression, but that how RBM10 autoregulation is achieved is cell type‐specific.

All of these very recent findings demonstrate that RBM10 can autoregulate its own alternative splicing, and expression at the mRNA and/or protein level. It is not unusual for an RBP to be able to regulate its own expression, especially given its RNA binding abilities. For instance, members of the Fox family, which includes *RBM9* (*FOX‐2*), can auto‐regulate the alternative splicing of *FOX* mRNA.^48^ In fact, FOX proteins promote exon exclusion during *FOX* alternative splicing, resulting in a translated variant with reduced RNA binding capabilities.^49^ This exclusion variant represses FOX's influence on the alternative splicing of other genes, thus FOX proteins not only autoregulate their alternative splicing, but also their function.^48^ In addition, an antisense transcript of *RBM5* can negatively affect full‐length *RBM5* expression, as well as alter levels of other *RBM5* alternative splice variants.^50^, ^51^ The area of RBM10 autoregulation is only beginning to be explored and many questions remain, including if RBM10v2 protein can also regulate *RBM10* expression. Further studies in this area are necessary to better understand how the *RBM10* splice variant and isoform expression profiles in a given system are controlled.

### 3.3 Relationship between RBM10 and RBM5

*RBM10* is a member of an RBP family that includes *RBM5* and *RBM6*. Phylogenetic studies provide evidence that *RBM5* is the progenitor gene.^52^ *RBM5* maps to chromosome three at position 3p21.3 and was first cloned by Wei et al in 1996^53^ under the name *LUCA‐15*. The full‐length *RBM5* transcript is approximately 3 kb, divided into 25 exons, and codes for a protein of 815 amino acids (Figure 1B). To date, *RBM5* has been studied more comprehensively than *RBM10*, and has been established as a tumor suppressor gene.^23^ The level of amino acid homology between RBM5 and RBM10 is approximately 50%, with RBM10v1 sharing 49% identity with RBM5, and RBM10v2 sharing 53%.^23^ The nucleotide sequences in exons 4, 9, and 15 are particularly different between *RBM5* and *RBM10* and are the main cause of the variation between both proteins.^23^ Of note, these non‐homologous exons are located right before consensus functional motifs, suggesting similar functionality for *RBM5* and *RBM10*, but potentially different specificity for both RBPs.^54^, ^55^ A relationship between products from the two genes has been demonstrated in a variety of cell types. For instance, both positive and negative expression correlations have been noted: (1) in HEK293 human embryonic kidney cells, *RBM10* overexpression correlated with increased *RBM5* exon 6 exclusion and overall decreased *RBM5* mRNA levels, whereas *RBM10* knockdown correlated with a slight decrease in *RBM5* exon 6 exclusion and overall higher *RBM5* mRNA expression levels^29^, ^33^; (2) in SHSY5Y human neuronal cells, *RBM10* knockdown correlated with increased levels of *RBM5* protein^45^; (3) in H9c2 rat myoblast cells, *RBM5* knockdown correlated with decreased *RBM10* mRNA expression^56^; and (4) in GLC20 *RBM5*‐null small cell lung cancer cells, increased *RBM5* expression correlated with increased protein expression of specific *RBM10* splice variants.^21^ Of note, the first and fourth study described not only showed a correlation between *RBM5* and *RBM10* expression, but also direct interactions between them: (1) in HEK293 cells, RBM10 bound the *RBM5* intron 5 5′‐splice site and intron 6 3′‐splice site, influencing *RBM5* alternative splicing and ultimately leading to RBM5 AS‐NMD^33^; and (2) in GLC20 cells, RBM5 bound only one specific *RBM10* splice variant, and was associated with increased protein expression of RBM10v2.^21^ Considered together, these results demonstrate that relationships between RBM5 and RBM10 occur across cell types and species, highlighting their potential fundamental importance to the cell.

### 3.4 RBM10's pro‐transformatory functions may be RBM5‐dependent

The functional consequences of the relationship between RBM5 and RBM10 have yet to be fully grasped, but our group recently presented data and a working model that link RBM5 to the regulation of RBM10 function: in an *RBM5*‐null environment, the putative tumor suppressor RBM10 actually promoted transformation‐associated processes.^21^ The working model presented by Loiselle et al^33^ which describes this association, is (a) comprehensive, taking into account even the most recently published findings regarding RBM10, and (b) supported by data presented in another manuscript, which was published after submission of the Loiselle et al. manuscript, that demonstrated the autoregulatory functions for RBM10 described above.

Three recent gene expression studies involving various tumor types provide in vivo data supporting the suggestion that RBM10 promotes transformation in systems where *RBM5* is downregulated. Firstly, gene expression analysis of Cancer Genome Atlas (TCGA) samples from various tumor types showed that *RBM10* was significantly upregulated in many cancer types, while *RBM5* expression was significantly downregulated.^10^ Secondly, *RBM10* mutations in pancreatic ductal cancer correlated with a better 5‐year survival probability^14^ and *RBM5* mRNA and protein expression was significantly reduced in pancreatic cancers.^57^ Thirdly, *RBM10* expression correlated with increased disease aggressiveness in metastatic melanomas^58^ and the RBM5 promoter region was found to be significantly mutated in metastatic melanomas,^59^ meaning that *RBM5* expression is likely compromised in this type of cancer. Taken together, these in vivo studies and our in vitro studies suggest that *RBM10* expression promotes transformation in *RBM5*‐reduced environments. The nature of this relationship between RBM5 and RBM10 has thus begun to be elucidated; more studies are required, however, to completely understand this association.

### 3.5 Clinical importance of RBM10

The clinical importance of understanding RBM10 function and regulation is highlighted in situations where *RBM10* expression is disrupted. For instance, *RBM10* mutations are the cause of TARP syndrome.^6^, ^8^ This condition is characterized by many developmental abnormalities, particularly craniofacial deformities such as cleft palate, glossoptosis (tongue displacement) and micrognathia (undersized jaw), which can cause difficulty eating and breathing.^7^ Sadly, usually due to various heart conditions associated with the disease, the affected children die before, or soon after, birth.^6^, ^7^, ^8^ If significant medical attention is provided, however, there have been reports of children with TARP syndrome living up to three years.^8^ These cases have permitted doctors to observe other phenotypic consequences of embryonic *RBM10* mutations, including chronic lung disease, visual impairment, significant intellectual disability and an inability to eat or sit independently.^8^, ^60^ The severe impact of *RBM10* mutations in these children strongly suggests a critical role for *RBM10* in fetal development. This is supported by in vivo and in vitro findings showing *RBM10* expression to be: (1) regulated during rat skeletal and cardiac muscle cell differentiation^61^; (2) regulated both temporally and spatially during murine midgestation embryo development^6^; (3) a regulator of mouse embryonic stem cell proliferation and differentiation^22^; and (4) able to influence the alternative splicing of *SMN2*.^32^ The latter could be of clinical relevance to patients with spinal muscular atrophy who have a homozygous deletion of *SMN1*, and thus rely on their *SMN2* gene for all SMN protein.

*RBM10* is also mutated in select cancer types. For instance, *RBM10* was found to be truncated in approximately 7% of lung adenocarcinomas,^9^, ^10^, ^62^ with this mutation rate increasing to 21% in invasive lung adenocarcinomas.^13^ *RBM10* mutations have also been identified in pancreatic intraductal papillary mucinous neoplasms,^12^ pancreatic ductal adenocarcinomas,^14^ colorectal cancers,^11^ and fatal forms of non‐anaplastic thyroid cancer.^15^ Despite this, in a mutational screen of 441 tumors of various cancer types including pancreatic and lung adenocarcinomas, only one breast and one prostate cancer sample, respectively, had an *RBM10* mutation.^63^ The importance of functional *RBM10* in regards to the transformed state, therefore, remains to be determined.

## 4 CONCLUSION

RBM10‐related studies are rapidly gaining momentum. Even in the past year, knowledge regarding RBM10 has significantly expanded; it is now established that RBM10 has a number of alternative splice variants, is autoregulated, and interacts with RBM5. There is thus an intricate system regulating *RBM10* expression and function which is only beginning to be revealed. Future studies in this area could include determining: (1) the function of each *RBM10* splice variant; (2) the mechanism of action of each *RBM10* splice variant (eg, if all splice variants have alternative splicing capabilities); (3) which *RBM10* splice variants are posttranscriptionally modified; and (4) the factors that regulate *RBM10* splice variant expression. More studies are also required to fully understand the nature of the relationship between RBM5 and RBM10, how their interactions are regulated, and how these may vary between cell types and disease states. In addition, future work could include examining if/how expression of *RBM6*, another *RBM5* family member, influences *RBM10* expression and function. Interestingly, knockdown of *RBM5, RBM10*, and *RBM6* was necessary to demonstrate the ability of RBM5 to modulate *FAS* alternative splicing, suggesting a potential relationship between all three family members.^64^

In sum, by considering the various aspects of RBM10 regulation that are described in this review, in future RBM10‐related experimental endeavors, a much clearer insight into RBM10 function, and influence on disease states, will be gained. Ultimately, this may lead to the development of novel and effective treatment options for these diseases.
